# Poultry Culling and Campylobacteriosis Reduction among Humans, the Netherlands

**DOI:** 10.3201/eid1803.111024

**Published:** 2012-03

**Authors:** Ingrid H.M. Friesema, Arie H. Havelaar, Paul P. Westra, Jaap A. Wagenaar, Wilfrid van Pelt

**Affiliations:** National Institute of Public Health and the Environment (RIVM), Bilthoven, the Netherlands (I.H.M. Friesema, A.H. Havelaar, W. van Pelt);; Utrecht University, Utrecht, the Netherlands (A.H. Havelaar, J.A. Wagenaar);; Product Boards for Livestock, Meat and Eggs, Zoetermeer, the Netherlands (P.P. Westra);; Central Veterinary Institute, Lelystad, the Netherlands (J.A. Wagenaar);; World Health Organization Collaborating Centre for Reference and Research on Campylobacter, Utrecht (J.A. Wagenaar);; World Organisation for Animal Health Reference Laboratory for Campylobacteriosis, Lelystad (J.A. Wagenaar)

**Keywords:** campylobacter, campylobacter infections, campylobacteriosis, human, poultry, epidemiologic factors, environment, culling, bacteria, the Netherlands

## Abstract

In the Netherlands in 2003, an outbreak of avian influenza in poultry resulted in extensive culling, especially of layer hens. Concurrently, human campylobacteriosis cases decreased, particularly in the culling area. These observations raise the hypothesis that *Campylobacter* spp. dissemination from poultry farms or slaughterhouses might contribute to human campylobacteriosis.

In the Netherlands during March–May 2003, an outbreak of avian influenza (H7N7) virus among poultry led to the culling of >30 million birds (*1*). The outbreak, and thus the culling, was confined to a relatively small area of 50 × 30 km in the center of the country (*2*). A few years after the avian influenza outbreak, it became apparent that the incidence of campylobacteriosis among humans had decreased during 2003 and that the extent of this decrease varied by region. Because the avian influenza outbreak strongly affected the poultry industry in 2003, a link was suspected.

## The Study

In the Netherlands, the laboratory surveillance network for gastroenteric pathogens was started in 1987 and now consists of 15 regional public health laboratories. In April 1995, *Campylobacter* spp. were included in surveillance. Each laboratory reported the number of all first isolates of pathogens weekly to the Department of Epidemiology and Surveillance at the National Institute for Public Health and the Environment (RIVM). For 2002 through 2004, prospective weekly estimates of the expected frequency of campylobacteriosis cases and 99.5% tolerance levels were calculated by using the Farrington algorithm, based on weekly surveillance data for the preceding 5 years, and linear interpolation of the observed frequencies per year (1995–2008) (*3*,*4*). Incidence rates were calculated by taking the area covered by the surveillance network into account (*4*).

Campylobacteriosis incidence in the Netherlands decreased from 46.4 patients per 100,000 inhabitants in 1996 to 38.7 in 1999 and increased thereafter to 44.3 in 2001 and 40.8 in 2002. In 2003, incidence decreased to 33.3 per 100,000 inhabitants, and during 2004–2008, it increased again to 40.0–43.8.

**Figure 1:**
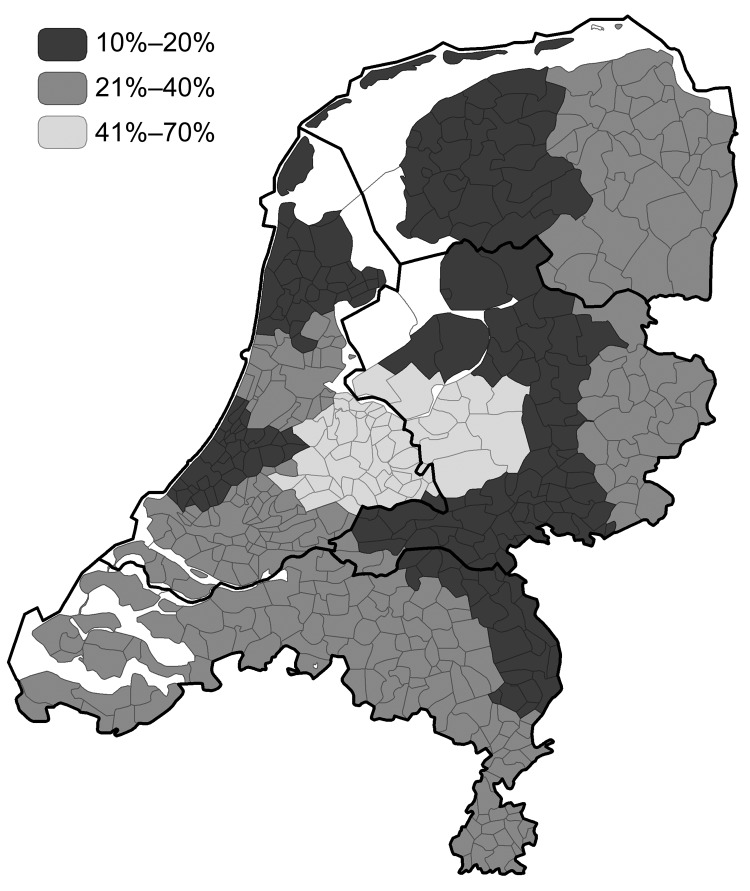
Regional reduction of campylobacteriosis (March–December 2003) following the Public Health Laboratory regions borders in the Netherlands, with the outlines of the 4 clusters of provinces.

**Figure 2:**
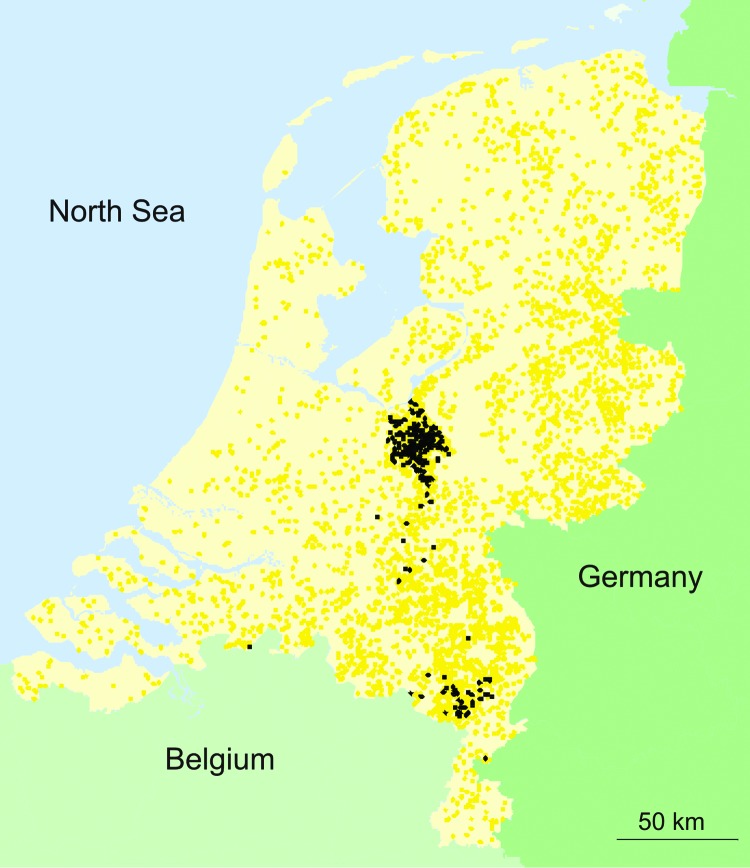
Locations of all 5,360 commercial poultry farms in the Netherlands (*2*). Black dots indicate farms that were infected during the 2003 epidemic of avian influenza; yellow dots indicate farms that were not infected.

**Figure A1:**
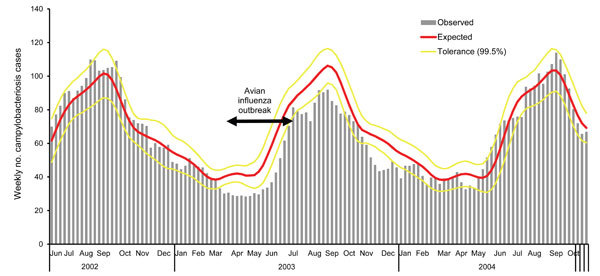
Weekly number of cases of campylobacteriosis among humans, the Netherlands, 2002–2004.

In March 2003, a 30% reduction of reported campylobacteriosis cases in the Netherlands was noted. In December 2003, a 19% reduction was noted (Figure A1). From March through December 2003, levels of reduction varied markedly among public health laboratories, 10%–70%; the largest reduction occurred in the central region of the country, where the culling took place (Figure 1, Figure 2) (*2*). Overall, the percentages of cases reported by the laboratories in culling areas were 44%–50% less than expected during May–December 2003.

In the poultry culling area, 1 large slaughterhouse (2 locations) and 1 smaller slaughterhouse, which together accounted for 15% of the national slaughter capacity for broiler chickens, had to be closed during the culling (March–June). Information about poultry purchases was gathered through registration of the food products bought by and interviews with a random sample of 6,000 households, comprising ≈13,400 persons, by GfK Panel Services Benelux (Dongen, the Netherlands) (*5*). The Product Boards for Livestock, Meat and Eggs provided sales data for poultry meat on the national level and stratified by 4 regions.

Comparison of broiler meat purchases during 2002–2003 (Table) indicated a national reduction in sales during March–October 2003; the reduction was greatest during May–June (−9%). The regional reduction never exceeded –12% and was largest in areas roughly overlapping or near the culling area. By 2004, sales had returned to normal (85,165 kg, data not shown).

**Table:** Changes in broiler meat sales, by region, the Netherlands, 2002–2003

Region*	Sales × 1,000 kg, 2002/2003	Change, %					
		Jan–Feb	Mar–Apr	May–Jun	Jul–Aug	Sep–Oct	Nov–Dec
Entire country	84,128/81,137	1	−6	−9	−5	−2	4
Mideastern region†	17,435/16,582	−3	−7	−12	−5	−2	0
Western + middle regions‡	40,546/38,351	−2	−6	−11	−6	−4	−3
Northeastern region§	7,022/7,135	3	−3	−8	0	6	12
Southern region¶	19,125/19,068	−2	−2	−2	2	2	2

*Most culling was conducted in Gelderland and Utrecht Provinces. †Flevoland, Gelderland, Overijssel Provinces. ‡Noord Holland, Zuid Holland, and Utrecht Provinces. §Groningen, Friesland, and Drenthe Provinces. ¶Zeeland, Noord Brabant, and Limburg Provinces.

## Conclusions

Consumption of poultry and direct contact with poultry are generally accepted as dominant risk factors for sporadic *Campylobacter* spp. infections among humans (*6*,*7*). In the Netherlands, the strongest reduction in campylobacteriosis cases occurred in the laboratory service areas overlapping the culling area and the areas where the slaughterhouses were closed. Also, sales of poultry meat dropped most in these areas, although not proportional to the reduction in campylobacteriosis, and recovered quickly after June; the reduction in campylobacteriosis occurred at least up to the end of the year. Moreover, culling was mainly among layer hens (54%) and only 8% among broilers. In the Netherlands, meat from spent hens (layer hens that are no longer economically productive) is not consumed as fresh meat.

Environmental pathways of human *Campylobacter* spp. infection remain less understood (*7*) and might play a major role in rural areas (*8*,*9*). These pathways remain to be clarified, although some studies have implicated aerosols and flies as vectors for environmental transmission (*10*–*12*). *Campylobacter* spp. have been detected in the air up to 30 m downwind of and in puddles near broiler houses (*13*). A US study among chicken catchers and poultry plant workers at 1 plant found colonization with *Campylobacter* spp. among 41% and 63% of these persons, respectively (*14*). Surprisingly, 9 community members who lived near, but did not work at, the US plant had positive *Campylobacter* spp. test results.

In Belgium in 1999, the availability of poultry meat was greatly reduced because of dioxin-contaminated feed components (*15*). All poultry meat and eggs from Belgium were withdrawn from the market, which resulted in a 40% decrease in campylobacteriosis cases for 2 weeks after the withdrawal. Two weeks after sale of these products resumed, incidence returned to previous rates, although poultry production took 7 weeks longer to regain levels similar to those of the year before. In the Netherlands, the reduction in campylobacteriosis cases lasted longer. The situations in the Netherlands and Belgium also differed at other points. In the Netherlands, culling was conducted in a relatively small area, at farms under strict biosecurity measures, and was followed by intensive cleaning and disinfection of the farms and an extended period when farms were empty. In Belgium, the poultry came from farms throughout the country and were slaughtered according to routine procedures before disposal of carcasses or processed meat. Furthermore, in the Netherlands, sales of broiler meat decreased by <12%, whereas in Belgium, 100% of broiler meat was withdrawn from the market.

In this retrospective study, measures of environmental dissemination of *Campylobacter* spp. were lacking. The use of aggregated data makes it impossible to prove a causal link between the culling of poultry and the decrease in campylobacteriosis incidence. Nevertheless, on the basis of the combined information, we hypothesize a relationship between the reduced environmental contamination by poultry farms and slaughterhouses and the reduced number of campylobacteriosis cases in humans in the same region. Because slaughterhouses were closed and disinfected farms were empty or closed for everyone except attendants under strict hygiene measures, a temporal, lower environmental load of *Campylobacter* spp. was probably achieved. We are not aware of any other events in this period that might explain the regional and temporary decrease in campylobacteriosis incidence. However, unobserved effects, such as improved kitchen hygiene resulting from regional consumers’ awareness of a link between poultry meat and infectious diseases, are also possible explanations.

Our hypothesis of secondary exposure to *Campylobacter* spp. through dissemination from poultry farms or slaughterhouses has public health implications. Even if poultry meat at retail is free of *Campylobacter* spp., campylobacteriosis could occur earlier through exposure during production; thus, control should start at this step of the food chain. More research, including microbiological, analytical, and risk assessment studies, needs to be done to prove or disprove the role of dissemination in the spread of *Campylobacter* spp. and to clarify the possible mechanisms of environmental transmission.
